# The Protective Role of Carnosic Acid against Beta-Amyloid Toxicity in Rats

**DOI:** 10.1155/2013/917082

**Published:** 2013-10-24

**Authors:** H. Rasoolijazi, N. Azad, M. T. Joghataei, M. Kerdari, F. Nikbakht, M. Soleimani

**Affiliations:** ^1^Cellular & Molecular Research Center, Iran University of Medical Sciences, Hemmat Highway, Tehran, Iran; ^2^Department of Anatomy, School of Medicine, Iran University of Medical Sciences, Hemmat Highway, Tehran, Iran; ^3^Department of Anatomy, School of Medicine, Tehran University of Medical Sciences, Pursina Avenue, Tehran, Iran; ^4^Department of Physiology, School of Medicine, Iran University of Medical Sciences, Hemmat Highway, Tehran, Iran

## Abstract

Oxidative stress is one of the pathological mechanisms responsible for the beta- amyloid cascade associated with Alzheimer's disease (AD). Previous studies have demonstrated the role of carnosic acid (CA), an effective antioxidant, in combating oxidative stress. A progressive cognitive decline is one of the hallmarks of AD. Thus, we attempted to determine whether the administration of CA protects against memory deficit caused by beta-amyloid toxicity in rats. Beta-amyloid (1–40) was injected by stereotaxic surgery into the Ca1 region of the hippocampus of rats in the Amyloid beta (A**β**) groups. CA was delivered intraperitoneally, before and after surgery in animals in the CA groups. Passive avoidance learning and spontaneous alternation behavior were evaluated using the shuttle box and the Y-maze, respectively. The degenerating hippocampal neurons were detected by fluoro-jade b staining. We observed that beta-amyloid (1–40) can induce neurodegeneration in the Ca1 region of the hippocampus by using fluoro-jade b staining. Also, the behavioral tests revealed that CA may recover the passive avoidance learning and spontaneous alternation behavior scores in the A**β** + CA group, in comparison with the A**β** group. We found that CA may ameliorate the spatial and learning memory deficits induced by the toxicity of beta-amyloid in the rat hippocampus.

## 1. Introduction

The depositions of amyloid *β* protein (A*β*) in the extracellular neuritic plaques, neurofibrillary tangles containing hyperphosphorylated tau protein in the neurons of the hippocampus and other parts of the cortex resulting in brain atrophy, are the most important neuropathological features associated with Alzheimer's disease (AD) [1–3]. An insidious onset of memory deterioration, progressive cognitive impairment, and behavioral disturbances are known to be important symptoms in AD [4]. The A*β* cascade hypothesis, which was developed in the early 1980s, shows that in the first phase of this disease, the deposition of amyloid plaques may affect cognition [5]. Increased permeability of the cell membranes, apoptosis, inflammatory reactions, and free radical damage are among the mechanisms that underlie A*β* neurotoxicity [6]. It has recently been accepted that oxidative stress also plays an important role in the pathogenesis of Alzheimer's disease [7].

Antioxidants can protect against the oxidative stress damage in different ways, including the inhibition of reactive oxygen species (ROS) formation [8].

Carnosic acid (CA), an important polyphenolic antioxidant, has been identified in *Rosmarinus officinalis* (rosemary plant) [9]. It is a lipophilic antioxidant with the ability to prevent lipid peroxidation and biological membrane disruption by scavenging oxygen hydroxyl radicals and lipid peroxyl radicals [10].

Additionally, it has been shown that CA could induce the transcriptional activation of antioxidant phase 2 enzymes such as electrophilic compounds. Thus, this type of neuroprotection could have beneficial effects in chronic neurodegenerative diseases like Parkinson's and Alzheimer's [11]. Our group has previously reported that CA protects the hippocampal neurons and decreases cellular death in an animal model of Alzheimer's disease [12].

Therefore, the present study aims to evaluate the protective effects of carnosic acid on cognitive impairment against the neurotoxicity induced by A*β* in the rat hippocampus.

## 2. Materials and Methods

### 2.1. Materials

A*β*-protein fragment (1–40) and carnosic acid were purchased from Sigma Chemical Co. (Saint Louis, MO, USA) and A.G. Scientific Co. (San Diego, CA, USA), respectively. Fluoro-jade b was purchased from Millipore (Billerica, MA, USA). A*β* (1–40) was dissolved in deionized water to a final concentration of 1.5 nmol/*μ*L and stored at −70°C before use. CA was dissolved in DMSO and stored at −20°C before use. Immediately prior to injection, PBS was added to CA + DMSO (PBS/DMSO: 10/1).

### 2.2. Animals

The male Wistar rats (Pasteur's Institute, Tehran, Iran) (*n* = 42) weighing 240–280 g that were used in this study were housed in the animal lab of the Iran University of Medical Sciences. The animals were maintained in laboratory cages (3 animals/cage) under a 12 h light/dark cycle, at a room temperature of 21 ± 2°C, and they had free access to food and water.

All the animal procedures were approved by the Animal Care Committee of the Chancellor for Research of the Iran University of Medical Sciences (Tehran, Iran), and all possible steps were taken to stay away from animals' suffering at each stage of experiments.

The animals were divided into six groups: control, vehicle, sham surgery, carnosic acid (CA), Amyloid beta (A*β*), and carnosic acid + A*β* (A*β* + CA). Animals in the A*β* groups were administered 1.5 nm/*μ*L beta-amyloid (1–40) in the Ca1 region on both sides of the hippocampus. Animals in the CA groups received 1 mL of CA solution (CA: 10 mg/kg) intraperitoneally, 1 hour prior to surgery. This treatment (CA: 3 mg/kg) continued a couple of hours after surgery and, subsequently, each afternoon for up to 12 days.

### 2.3. Methods

#### 2.3.1. Surgical Procedure

Animals were first anesthetized by the intraperitoneal (IP) injection of ketamine (100 mg/kg) and xylazine (20 mg/kg) and then positioned on a stereotaxic apparatus (Stoelting Co., USA). The injections were delivered through a 5 *μ*L Hamilton syringe at the level of the hippocampus, as following coordinates: antero-posterior, −3.8 mm; lateral, ±2.6 mm from bregma and −2.8 mm ventral from the dura. Each injection lasted for 5 minutes, and the needle was kept in place for an additional 5 minutes before being slowly withdrawn.

12 days after surgery, behavioral tests were performed on the animals in the animal lab of the Iran University of Medical Sciences.

#### 2.3.2. Passive Avoidance Task

The passive avoidance task was performed using the shuttle box to study the learning memory status in rats. The shuttle box consists of two equally sized compartments, with a guillotine (execution) door and a grid floor for the delivery of an electric foot shock. An electric light bulb illuminates one compartment, while the other remains in the dark. During the training session, the animals were individually placed in the light chamber, facing away from the execution door. When the animal entered the darkened compartment, the door was quietly lowered and a 0.5 mA foot shock was applied for 2 seconds through the grid floor. During the test session, the animal was placed in the light compartment once more, to enter the dark compartment, but the foot shock was not applied. The latency to step through was recorded [13]. Time latency was recorded up to a maximum of 300 seconds. 

#### 2.3.3. Y-Maze Spatial Memory

Spontaneous alternation behavior was performed using the Y- maze apparatus to study short-term spatial memory in rats. The Y-maze apparatus, made of gray Plexiglas and covered with a black paper, is shaped like a Y, with three identical arms with an angle of 120° between each pair of arms [14]. Each arm is 40 cm long, 30 cm high, and 15 cm wide. The arms come together in a central area to form an equilateral triangle that is 15 cm at its longest axis. Each animal was set out at the end of one arm and was then allowed to move freely inside the maze in an 8-minute session. When the base of the animal's tail was completely placed in the arm, each arm entrance was recorded visually (e.g., ABC, BBC, CBA, ABB, C). An alternation was defined as successive entries into the different three arms on overlapping triplet sets (i.e., ABC, CBA) [15]. The percentage of spontaneous alternation behavior was calculated as the ratio of actual to potential alternations as follows: Percent Alternation = {Actual Alternation (i.e., ABC, CBA: 6)/[Maximum Alternation (i.e., ABCBBCCBAABBC: 13) − 2] × 100 = (6/11) × 100 = 54.54% [16].

#### 2.3.4. Fluoro-Jade b Staining

Fourteen days following surgery, when the behavioral tests were conducted, rats were perfused with 4% paraformaldehyde in 0.1 M phosphate buffer (pH 7.4), and then the brains were extracted and placed in the same solution. The paraffin slides were mounted on gelatin-coated slides and stained with fluoro-jade b, a fluorescent staining technique for the detection of neuronal degeneration. The staining protocol was conducted as described by Schmued, LC (2000). Briefly, the sections of the brain were immersed in xylene and then placed in a solution containing 1% sodium hydroxide in 80% alcohol, followed by 2 min in 70% alcohol and 2 min in distilled water, and then the slides were transferred to a solution of 0.06% potassium permanganate for 10 min. Following rinsing in distilled water, the slides were placed in the fluoro-jade b staining solution (0.0004%) for about 20 min. Finally, the dry slides were cleared by immersion in xylene and mounted in water-free mounting medium, DPX, and a cover slip was placed on top. The sections were inspected with a 40x objective, using the FITC filter for the observation of neuronal degeneration [17].

Data were expressed as mean ± standard error of the mean (S.E.M.) and analyzed by the SPSS statistical software package (version 17). One-way ANOVA was used to analyze the difference between groups. Post hoc between-group comparisons were done using least square difference (LSD).

## 3. Results

Because there were no significant differences in the results between control, vehicle, and sham surgery groups, the results of these three groups are pooled together and shown as control group only.

### 3.1. Behavioral Results

A significant decrease in the passive avoidance learning and spatial Y-maze alternation scores was observed in the A*β* group, in comparison with the A*β* + CA group.

#### 3.1.1. Passive Avoidance Task

**Figure 1:**
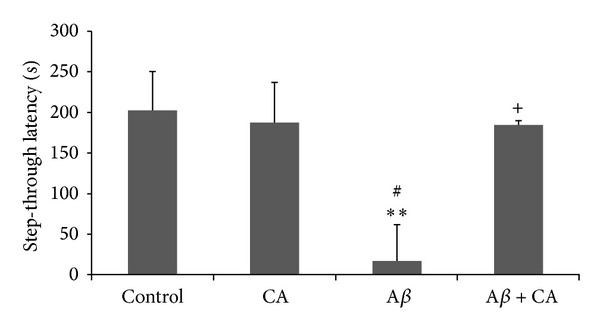
Step-through latency in the experimental groups (control, CA: carnosic acid, A*β*: Amyloid beta, and A*β* + CA: carnosic acid + A*β*) (mean ± SEM): **P < 0.01 compared to the control; ^#^ *P* < 0.05 compared to the CA group; ^+^ *P* < 0.05 compared to the A*β* group.

As shown in Figure 1, there is a significant decrease in the step-through latency in the A*β* group as compared to the control and the CA group (*P* < 0.01). Additionally, there is a significant increase in the step-through latency in the A*β* + CA group as compared to the A*β* group (*P* < 0.05). The mean step-through latency for the control, CA, A*β*, and A*β* + CA groups was 202.1 ± 48.5, 187.4 ± 44.3, 18 ± 4.9, and 184.8 ± 50.5, respectively.

#### 3.1.2. Spatial Y-Maze Memory

**Figure 2:**
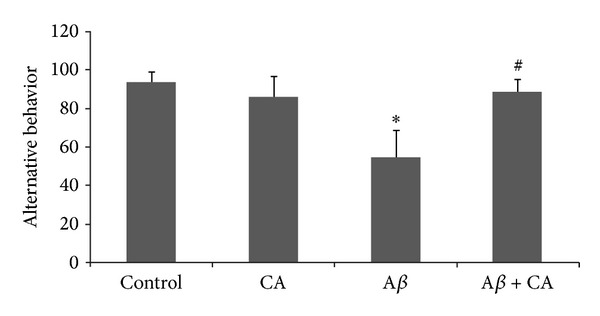
The percent of alternation behavior in the experimental groups (control, CA: carnosic acid, A*β*: Amyloid beta, and A*β* + CA: carnosic acid + A*β*) (mean ± SEM): *P < 0.05 compared to the control group and ^#^ *P* < 0.05 compared to the A*β* group.

As shown in Figure 2, the mean percent alternation behavior for the control, CA, A*β*, and A*β* + CA groups was 93.6 ± 5.3, 86 ± 14.3, 54.3 ± 6.1, and 89 ± 9.7, respectively. Thus, a significant decrease in the percent alternation behavior was observed in the A*β* group as compared to the control group (*P* < 0.05). Additionally, a significant increase in the percent alternation behavior was observed in the A*β* + CA group in comparison with the A*β* group (*P* < 0.05).

### 3.2. Fluoro-Jade b Staining

**Figure 3:**
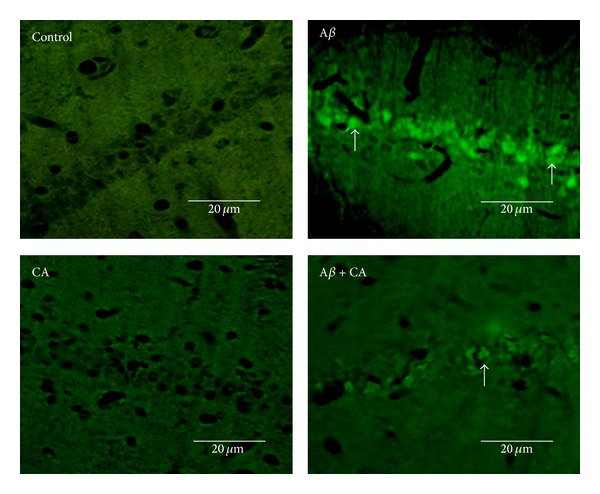
Fluoro-jade b staining in the Ca1 area of the hippocampus in the experimental groups (control, CA: carnosic acid, A*β*: Amyloid beta, and A*β* + CA: carnosic acid + A*β*). The white arrows show the fluorescent positive neurons in the Ca1 region of the hippocampus (400x).

Fluoro-jade b is recently used as a fluorescent marker for neuronal cell death and binds sensitively and specifically to the degenerating neurons. The positive neurons were observed with the green iridescence.  Figure 3 presents the fluoro-jade b staining in the Ca1 region of the hippocampus for the control, CA, A*β*, and A*β* + CA groups. As it is shown, there are so many degenerating neurons in the A*β* group, while there are fewer in A*β* + CA group, and there are not any positive neurons observed in control and CA groups.

## 4. Discussion

In Alzheimer's disease, lack of memory is one of the first symptoms to occur [18]. Therefore, in this study, we proposed a strategy against the *in vivo* A*β* (1–40) toxicity. The spatial and learning memories in rats were investigated using the Y-maze and shuttle box apparatus to compare their score changes in accordance with the protective role allocated to CA against A*β* toxicity.

Yan et al. showed that the injection of A*β* (1–42) impairs performance on the passive avoidance test (35% decreases in step-through latency) and the Y-maze test (19% decreases in alternation behavior) [19]. In another study, Rasoolijazi et al. found that the unilateral intrahippocampal injection of 4 *μ*L of 2 nmol/*μ*L A*β* (1–40) can reduce spatial memory and psychomotor coordination (PMC) in rats [20]. Additionally, work from our own laboratory recently showed that a bilateral intrahippocampal injection of 4 *μ*L of 1.5 nmol/*μ*L A*β* (1–40) can induce neuronal loss in the Ca1 region of the hippocampus [12].

Based on the results of the present study, we showed that the neuronal loss in the Ca1 region of the hippocampus induced by A*β* (1–40) may result in part from neuronal degeneration, as demonstrated by fluoro-jade b staining.

Studies showed that consequent to neural lesions, the decreased latency to step-through is caused by several kinds of cognitive deficits [21]. In this study, we showed that A*β* (1–40) can induce the impairment of scores in alternation behavior and passive avoidance tasks in rats.

Researchers observed that CA activates the Keap1/Nrf2 transcriptional factor, thereby protecting neurons from oxidative stress and excitotoxicity. In cerebrocortical cultures, CA-biotin accumulates in nonneuronal cells at low concentrations and in neurons at higher concentrations. Furthermore, based on the fact that CA can transfer into the brain, a single intraperitoneal injection of CA (1 mg/kg) 1 h prior to MCAO (middle cerebral artery occlusion) protects the brain against the toxic effects of the ischemia/reperfusion [11].

In addition to the antioxidant activity of carnosic acid [22], it has been reported to have several other beneficial effects, including chemoprotective effects in the presence of carcinogens, suppression of metalloproteinase-1 mRNA expression which is induced by UVA irradiation[23], anti-inflammatory effect [24], and neurotrophic activities [25]. Furthermore, Ninomiya et al. (2004) showed that oral administration of CA at a dose of 20 mg/kg/day for 14 days suppressed the increased epididymal fat and body weight gain in high fat diet-fed mice [26]. Additionally, CA can protect photoreceptors against light-induced oxidative damage and retinal dysfunction [27].

In this study, due to the passive shock avoidance learning test results, there is a 90.3% increase in the mean score of the A*β* + CA group as compared to the A*β* group. The results of the short-term spatial memory test demonstrated a 39% increase in the mean score in the A*β* + CA group as compared to the A*β* group.

## 5. Conclusion

Taken together, it is suggested that the administration of CA could significantly improve short-term spatial and learning memory scores following their impairment by A*β* toxicity. This protective role may be due to the antioxidant, anti-inflammatory, and neurotrophic activities of CA. Therefore, CA may be considered as a chemopreventive agent against neurodegenerative disorders like Alzheimer's disease.
